# Points in the Quarterly Report

**Published:** 1915-03-06

**Authors:** 


### DEVELOPMENTS AT THE LONDON HOSPITAL.

### Points in the Quarterly Report.

At the Quarterly Court held at the London Hospital on Wednesday last several matters of interest were reported, the principal of which we append below.

### Various War Items.

The hospital has been taking in wounded two or three days a week, and up to date has treated 2,000 British and Belgian soldiers. The War Office has decided definitely not to send out the London Hospital contingent which some months ago was offered to them by the hospital.

The Committee has given permission for a miniature rifle range to be installed in the far corridor of the out-patient basement.

The House Committee has given permission to visit the hospital to Mr. Lavery, the distinguished artist, who has been commissioned to paint a picture of the wards showing the wounded.

Mrs. Oppenheimer and Miss Barrett have presented ambulance cars for the use of the hospital for the duration of the war, and have kindly offered to pay for their maintenance.

Mr. Fielden has once more given evidence of his generosity by providing the hospital with a relief hospital at Huntingdon.

One of the surgeons, Mr. L. J. Austin, who was taken prisoner in Belgium right at the beginning of the war, has safely returned home after enduring considerable hardships.

At the special request of the War Office, the Committee has given permission to Mr. E. W. Morris, the Secretary, to accept the Hon. Secretaryship of the King George Hospital.

### The Nurses' Home and Nurses' Pensions.

The Committee has found it absolutely necessary to begin building a new Home for Nurses. This Home, which is being built in East Mount Street, will take 120 nurses, besides the usual accommodation for the domestic staff.

Mr. Evan Spicer, a member of the Committee, has made a gift to the hospital of £1,000, the interest of which is to be used to increase the pensions of sisters and nurses of over twenty years' standing, and whose pecuniary circumstances are, in the opinion of the matron and Committee, such as render their pension insufficient to enable them to live comfortably. This gift was made in memory of his daughter, Lilian Spicer, who died in September, 1914, while a probationer in the hospital.

### Statistics for the Year.

A specially interesting and satisfactory report was presented on the subject of anæsthetics, in which it was stated that, in 1914, 22,938 anæsthetics were given, and only four deaths under anæsthesia (none of which was the fault of the anæsthetist) resulted. This is an even better result than in 1913, when 22,080 anæsthetics were given, and seven deaths occurred.

In 1914, 18,310 in-patients were treated, as against 17,096 in 1913. The 1913 number was a record, and 1914 is still higher. The high figure of 1914 is partly due to the admission of wounded soldiers. In 1914 the number of out-patients treated was 170,491, as against 173,774 in 1913—a decrease of about 3,000. This is due, the report states, to the better working of the Insurance Act, some patients having been sent to their panel doctors instead of being treated at the hospital.

On October 16, 1914, there were 994 patients in the hospital, which is the largest number there has ever been. The rate of mortality on all cases was 7.11 per cent., which is the lowest that has ever been in the history of the hospital.

### Personalia.

The Hon. C. Hanbury-Tracy, a member of the London Hospital Committee, who has been out at the Front, has returned invalided.

Mr. Douglas Owen was congratulated on his knighthood.

